# Discovery of a novel orthototivirus-like virus in patients with vulvovaginal candidiasis

**DOI:** 10.3389/fcimb.2026.1779554

**Published:** 2026-03-19

**Authors:** Ziyuan Dai, Qiang Lu, Mingzhong Sun, Hongmei Chen, Yuchen Jiang, Taotao Yu, Zhipeng Wang, Yungang Wang, Rong Zhu

**Affiliations:** Department of Clinical Laboratory, Yancheng Third People’s Hospital, The Affiliated Hospital of Jiangsu Vocational College of Medicine, Affiliated Hospital 6 of Nantong University, Yancheng, Jiangsu, China

**Keywords:** novel, orthototiviridae, phylogenetic analysis, totivirus, vulvovaginal candidiasis

## Abstract

**Introduction:**

Vulvovaginal candidiasis (VVC) is a common fungal infection affecting women worldwide. Although the vaginal microbiome has been extensively studied, the diversity of viruses present in the vaginal microenvironment remains poorly characterized.

**Methods:**

Vaginal swab samples from patients diagnosed with VVC were subjected to viral metagenomic sequencing using an Illumina NovaSeq platform. Viral contigs were assembled, annotated, and screened against public databases. Genome organization, pairwise sequence identity, and phylogenetic relationships were analyzed to determine the evolutionary position of the detected virus.

**Results:**

Here, we identified a novel double-stranded RNA virus, tentatively named Vaginal-associated orthototivirus-like 1 (VAOTV-1), in vaginal swab samples from patients with vulvovaginal candidiasis. VAOTV-1 was represented by a partial genome sequence of 4,332 bp, encoding a complete RNA-dependent RNA polymerase (RdRp; 729 amino acids) and a partial capsid protein (CP; 532 amino acids). The encoded RdRp protein shared a maximum amino acid sequence identity of 47.43% with Totiviridae sp. isolate 22AP502 (GenBank accession no. XTJ93729.1), reported from Bandicota indica. In contrast, the CP showed no significant similarity to any sequences currently available in public databases, and BLASTn searches against the NCBI nucleotide database did not yield any significant matches. Phylogenetic analysis, together with the relatively low amino acid sequence identity to known members of the genus Totivirus within the family Orthototiviridae, suggests that VAOTV-1 represents a distinct and highly divergent orthototivirus-like lineage.

**Discussion:**

These findings indicate that VAOTV-1 represents a highly divergent orthototivirus-like virus and expands the known diversity of totiviruses detected in human-associated mucosal environments. This discovery highlights previously unrecognized viral diversity in the vaginal virome and provides new insights into viruses associated with vulvovaginal candidiasis.

## Introduction

The vagina harbors a complex microbial ecosystem that includes billions of microorganisms, such as bacteria, fungi, viruses, and archaea, whose roles in vaginal health and disease are not fully understood (Witkin and Forney, 2020; Honorato et al., 2024). Recent studies have made significant progress in characterizing the composition and structure of the vaginal microbiota, revealing changes in the vaginal microbiome during bacterial, fungal, or viral infections (Onderdonk et al., 2016; Chen et al., 2021; Li et al., 2023). Moreover, the vaginal bacterial microbiome has been shown to be associated with cervical disease progression (Mitra et al., 2016; Kyrgiou et al., 2017; Laniewski et al., 2020), and alterations in the vaginal eukaryotic virome have been reported in women with different grades of cervical lesions or cervical cancer (Li et al., 2023).

In addition to bacterial vaginosis, vulvovaginal candidiasis (VVC) is a common form of infectious vaginitis mainly caused by *Candida albicans*, affecting women across all age groups, with approximately 5-8% of patients developing recurrent episodes, known as recurrent VVC (Sun et al., 2023). Notably, previous studies have not identified significant alterations or atypical vaginal bacterial communities in women with frequent VVC compared with those without frequent episodes (Zhou et al., 2009). However, other studies have revealed highly variable patterns of the vaginal microbiome in patients with VVC (Liu et al., 2013); therefore, this issue remains unresolved.

The family *Orthototiviridae* comprises double-stranded RNA viruses with monopartite genomes of 3.9-5.6 kb in length, infecting a wide range of eukaryotic hosts, including protists, fungi, plants, and invertebrates. It currently includes only one recognized genus, *Totivirus* (https://ictv.global/report/chapter/orthototiviridae/orthototiviridae). However, because these viruses are generally present at low abundance and are infrequently detected, they have been largely overlooked in studies of the human vaginal microbiome. For example, among the 34 relevant studies surveyed by Honorato et al (Honorato et al., 2024), only one reported the detection of such a virus, which was classified at the time within the family *Totiviridae* and was later reassigned to the family *Orthototiviridae* following the 2023 taxonomic revision (Reyes-Proano et al., 2024; Simmonds et al., 2024).

Here, we identified a previously unreported RNA virus in vaginal swab samples collected from patients with VVC using next-generation sequencing. Analysis of its sequence features and phylogenetic relationships demonstrated that this virus represents a novel orthototivirus-like lineage, which we named Vaginal-associated orthototivirus-like 1 (VAOTV-1).

## Methods

### Study participants and ethical approval

Vaginal swab samples were collected from 12 women diagnosed with VVC at the Affiliated Hospital 6 of Nantong University in 2024. The diagnosis of VVC was based on clinical manifestations and microscopic examination of vaginal secretions. Individuals were excluded if they were pregnant, had received antibiotics within the previous month, were receiving immunosuppressive therapy, or had a history of cervical treatment or surgery. All specimens were obtained from the Department of Clinical Laboratory, anonymized prior to analysis, and the requirement for informed consent was waived by the institutional ethics committee. This study was approved by the Ethics Committee of the Affiliated Hospital 6 of Nantong University (Approval No. 2024-34), and all procedures were conducted in accordance with relevant ethical guidelines.

### Sample collection, processing, and sequencing

Vaginal swab specimens were collected by gynecologists during routine clinical examinations using a vaginal speculum. Sterile swabs were used to sample the anterior and posterior vaginal fornices as well as cervical secretions. Each swab was immediately transferred into a sterile tube and maintained at 4 °C. Prior to viral metagenomic analysis, the swab tips were submerged in 0.5 mL of Dulbecco’s phosphate-buffered saline (DPBS), followed by vigorous vortexing for 5 min and incubation at 4 °C for 30 min. After centrifugation at 15,000 × g for 10 min, the supernatants were collected and stored at -80 °C until downstream analysis.

For virome enrichment, approximately 45 μL of supernatant from each specimen was pooled and filtered through a 0.45 μm pore-size membrane (Millipore, Darmstadt, Germany) to remove host cells, bacteria, and large viral particles. The resulting filtrate was treated with DNase and RNase at 37 °C for 60 min to eliminate unprotected nucleic acids. Viral nucleic acids were subsequently extracted using the QIAamp MinElute Virus Spin Kit (Qiagen, Germany) according to the manufacturer’s instructions and eluted in DEPC-treated water supplemented with RNase inhibitors. Reverse transcription was performed using random hexamer primers (100 pmol) and reverse transcriptase (PureScript Enzyme, Vazyme, China), followed by second-strand DNA synthesis using Klenow fragment polymerase (New England BioLabs, USA). This workflow enabled the recovery of viral nucleic acids from both RNA and DNA viruses. A sequencing library was prepared using the TruePrep DNA Library Prep Kit (Vazyme, China) and sequenced on an Illumina NovaSeq 6000 platform to generate 150 bp paired-end reads.

Throughout the experimental workflow, stringent contamination-control measures were implemented to prevent contamination and nucleic acid degradation. Aerosol-resistant filter tips were used during all handling steps. All consumables in direct contact with nucleic acids, including microcentrifuge tubes and pipette tips, were certified DNase- and RNase-free. For negative controls, sterile ddH_2_O was processed in parallel with clinical samples under identical experimental conditions. Sequencing of the control library on the Illumina NovaSeq 6000 platform generated only a negligible number of reads.

### Metagenomic assembly and host read removal

Raw FASTQ files were quality-filtered using fastp v1.0.1 (Chen, 2025) with default settings. To minimize host contamination, the human reference genome (Homo sapiens, GCF_000001405.40) was downloaded from NCBI, and Bowtie2 v2.4.5 (Langmead and Salzberg, 2012) was used to map and remove potential host-derived reads. Paired-end reads were assembled using MEGAHIT v1.2.9 (Li et al., 2016) with default parameters.

### Identification of eukaryotic viral genomes

The assembled contigs were screened against a local non-redundant (nr) protein database downloaded in August 2025 using the BLASTx program implemented in DIAMOND v2.1.13 (Buchfink et al., 2021), and significant sequences with an E-value cutoff of < 10–^5^ were filtered. Taxonomic identification of the viral dataset was performed using TaxonKit (Shen and Ren, 2021) and the rma2info program within MEGAN7 (Gautam et al., 2022). Contigs annotated as eukaryotic viruses were manually inspected in Geneious Prime v2024.0.5 (https://www.geneious.com) and subsequently used as reference sequences for mapping clean reads using the default recommended parameters to obtain complete or nearly complete genomes. The resulting genomic sequences were screened for potential vector contamination using VecScreen (https://www.ncbi.nlm.nih.gov/tools/vecscreen).

### Annotation of viral genomes

Geneious Prime was used with the parameters: minimum size = 400, start codon = ATG, to predict potential open reading frames (ORFs). These ORFs were subsequently validated through comparison with similar viruses in the GenBank database. The annotations of these ORFs were assigned based on comparisons with the Conserved Domain Database (Wang et al., 2023).

### Nested PCR screening for orthototivirus

Prior to nested PCR screening, viral RNA was individually extracted from all 12 vaginal swab samples using the QIAamp Viral RNA Mini Kit (Qiagen, Germany) according to the manufacturer’s instructions. First-strand cDNA synthesis was performed using a commercial reverse transcription kit (Vazyme, China) in a 20 μL reaction volume following the manufacturer’s protocol. The reaction was carried out at 42 °C and terminated at 85 °C for 5 min. The resulting cDNA was directly used as template for nested PCR amplification. Based on conserved regions of the viral RNA-dependent RNA polymerase (RdRp) gene identified from the pooled metagenomic assembly, specific primers were designed to detect VAOTV-1 in individual vaginal swab samples. Primer design was performed using the Design New Primers function implemented in Geneious Prime^®^ 2024.0.5. Nested PCR was conducted under the following conditions: initial denaturation at 95 °C for 5 min; 31 cycles of denaturation at 95 °C for 30 s, annealing at 52 °C (first round) or 55 °C (second round) for 30 s, and extension at 72 °C for 40 s; followed by a final extension at 72 °C for 5 min. Premixed rTaq DNA polymerase (TaKaRa, China) was used for amplification. Primers are listed in **Supplementary Table 1**. A no-template control was included in all PCR runs. Samples were considered positive if a PCR product of the expected size (292 bp) was detected on a 2% agarose gel stained with YeaRed (YEASEN, China).

### Phylogenetic analysis

To elucidate phylogenetic relationships, sequences representing different groups of related viruses were downloaded from the GenBank database, together with sequences of proposed species pending ratification. Nucleotide or protein sequences were aligned using MUSCLE, as implemented in MEGA-12 (Kumar et al., 2018), and the resulting alignments were trimmed using trimAl v1.5 (Capella-Gutierrez et al., 2009). Phylogenetic trees were inferred using IQ-TREE v2.3.6 (Minh et al., 2020) with 1,000 bootstrap replicates (*-bb 1000*) under models selected by ModelFinder (*-m MFP*). Phylogenetic trees were visualized and edited using Interactive Tree Of Life (iTOL) (Letunic and Bork, 2024).

### Pairwise sequence identity analysis

Pairwise protein sequence identity comparisons between VAOTV-1 and related lineages were performed using the Sequence Demarcation Tool v1.3 (Muhire et al., 2014). Amino acid sequences of the corresponding viral proteins were aligned using the default settings, and pairwise identity values were calculated for all sequence combinations. The resulting identity matrices were used to assess genetic relatedness between VAOTV-1 and its closest relatives and to support taxonomic assignment.

## Results

### Overview of the vaginal virome

**Figure 1:**
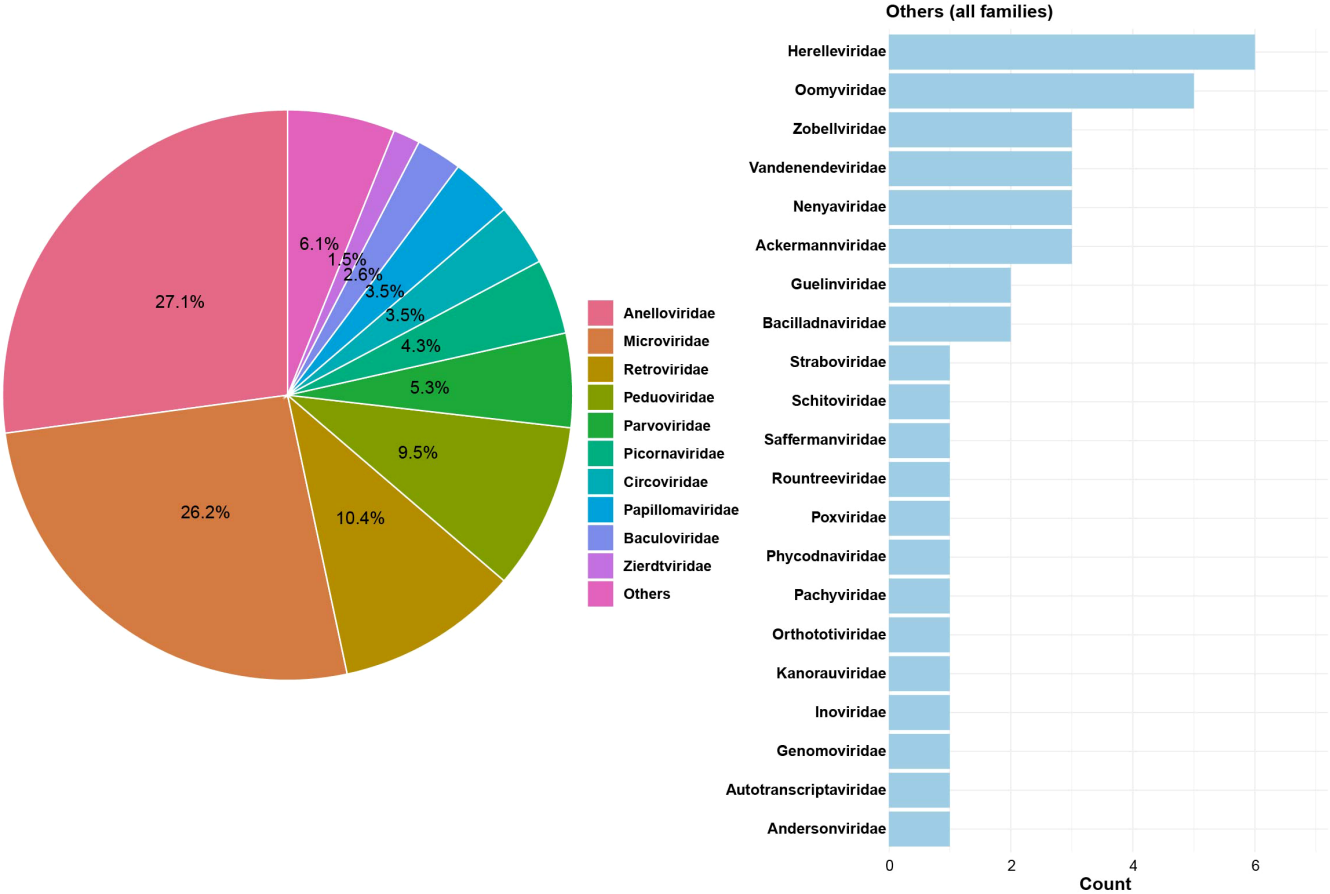
Composition of viral families in the metagenomic library and detailed breakdown of the “Others” category. The pie chart summarizes the relative composition of viral families, with the top 10 families shown individually and all remaining families grouped as “Others”. The bar chart presents the distribution of viral families included in “Others”, with bars indicating the number of contigs assigned to each family.

The vaginal metagenomic library yielded 17,802,393 clean reads after quality control and host read removal. *De novo* assembly of the clean reads generated a total of 233,307 contigs. Following similarity searches against the nr protein database, 656 contigs were taxonomically assigned as viruses. A total of 31 viral families were detected. *Anelloviridae* was the most abundant family, accounting for 27.1% of the total viral contigs, followed by *Microviridae* (26.2%), *Retroviridae* (10.4%), *Peduoviridae* (9.5%), *Parvoviridae* (5.3%), *Picornaviridae* (4.3%), and *Circoviridae* and *Papillomaviridae* (both 3.5%) (**Figure 1** and **Supplementary Table 2**).

In addition to bacteriophages, several eukaryotic viral families that are considered to have potential relevance to human health, including *Anelloviridae (*Kaelin et al., 2022), *Papillomaviridae (*Li et al., 2023), and *Circoviridae (*Wu et al., 2024), were identified in this study. The majority of contigs assigned to these families shared more than 90% amino acid sequence identity with known viruses, indicating close relatedness to previously characterized viral taxa. In contrast, one contig assigned to the realm *Riboviria* shared less than 50% amino acid sequence identity with any sequences currently available in public databases. The detection of a putative novel eukaryotic virus with such low sequence similarity in human-derived samples appears to be relatively uncommon. Accordingly, this contig was selected for further analysis to clarify its genomic characteristics and evolutionary relationships.

### Identification of a novel orthototivirus

**Figure 2:**
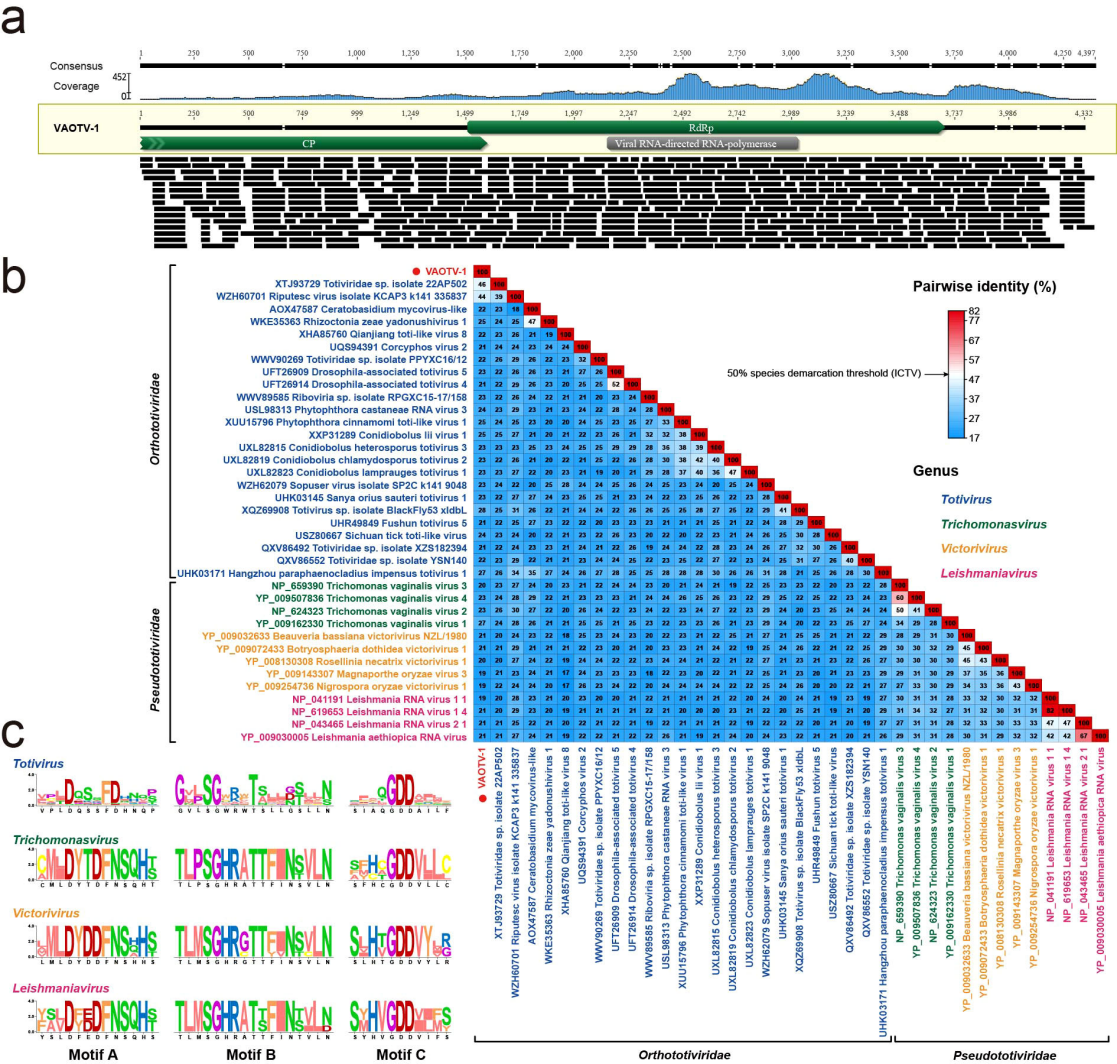
Genome organization of VAOTV-1 and sequence demarcation analysis of its closest RdRp-related virus. **(a)** Genome organization of VAOTV-1. The viral genome contains two overlapping ORFs. ORF1 encodes a partial putative CP, whereas ORF2 encodes the RdRp. The relative positions, orientations, and lengths of the ORFs are indicated. **(b)** SDT analysis based on RdRp amino acid sequences showing pairwise identity between VAOTV-1 and its closest related viruses. The color scale indicates percentage amino acid identity. **(c)** Alignment-derived consensus sequences of the conserved RdRp motifs **(A–C)** from representative viruses of the genera *Totivirus*, *Trichomonasvirus*, *Victorivirus*, and *Leishmaniavirus*. Motif regions were obtained from the multiple alignment used for the SDT-based pairwise identity analysis.

This viral contig was recovered through manual read mapping using Geneious Prime and was designated VAOTV-1. Based on similarity searches against the nr protein database, this sequence was initially considered a member of the family *Orthototiviridae*. The near-complete genome of VAOTV-1 is 4,332 bp in length, lacking a short segment at the 5′ end, with a GC content of 43.4% and a nucleotide composition of 30.1% A, 26.6% T, 20.6% G, and 22.8% C. A total of 4,113 clean reads were mapped to the VAOTV-1 genome, corresponding to a theoretical mean sequencing coverage of approximately 142× (**Figure 2a**).

**Figure 3:**
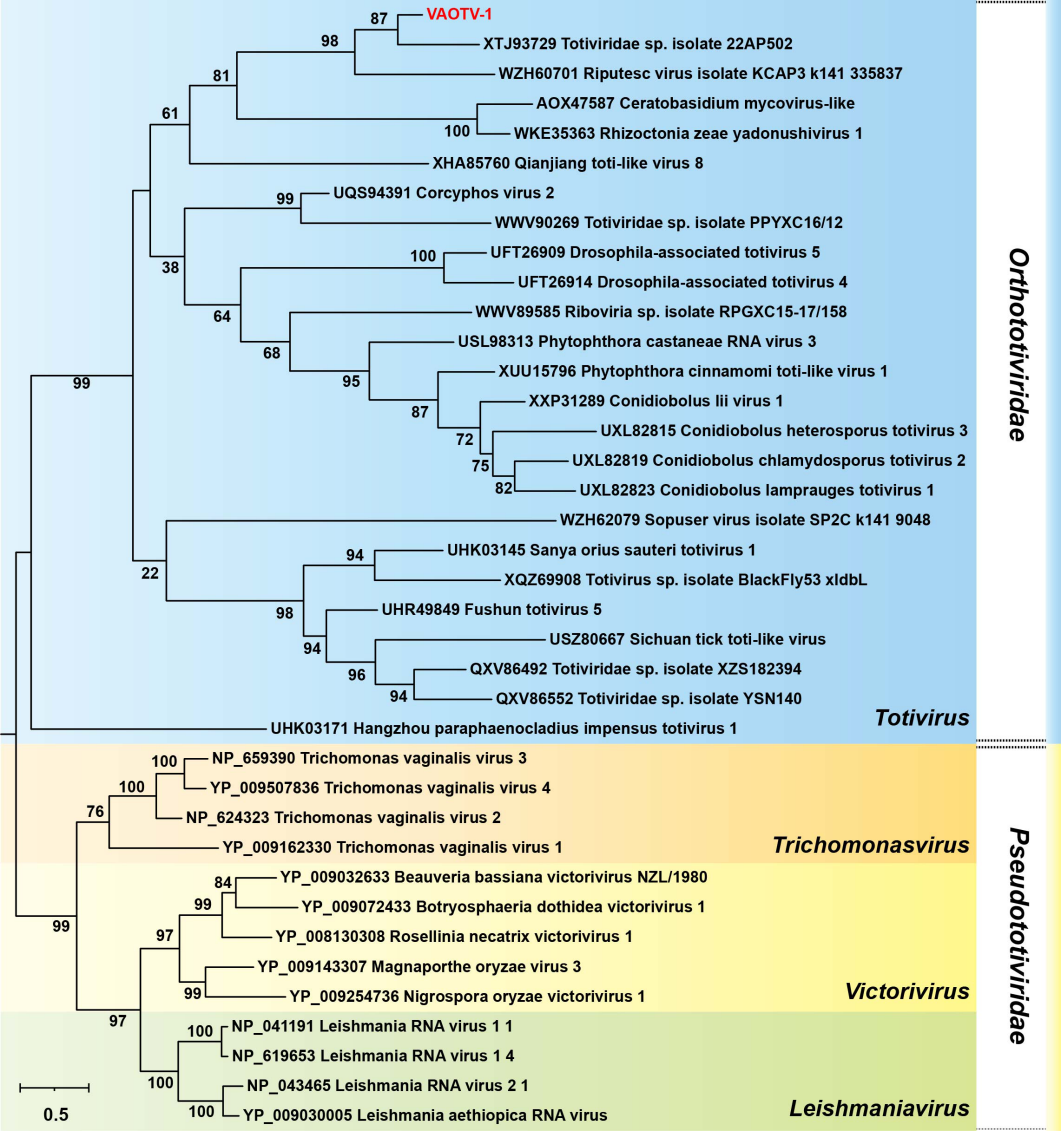
Evolutionary relationships of VAOTV-1 and other members of the families *Orthototiviridae* and *Pseudototiviridae*. The phylogenetic tree was inferred from RdRp amino acid sequences. Bootstrap support values are shown at the nodes. VAOTV-1 is highlighted in red.

The genome of VAOTV-1 contains two overlapping ORFs. ORF1 encodes a partial putative CP of 532 amino acids, whereas ORF2 encodes a putative RdRp of 729 amino acids. The RdRp protein shared a maximum amino acid sequence identity of 47.43% with *Totiviridae* sp. isolate 22AP502 (GenBank accession no. XTJ93729.1), which was isolated from *Bandicota indica*. In contrast, the CP showed no significant similarity to any sequences currently available in public databases. In addition, BLASTn searches against the NCBI nucleotide database did not yield any significant matches. To further evaluate the evolutionary relatedness of VAOTV-1, pairwise amino acid sequence identities of the RdRp region were calculated using the SDT based on BLASTx-matched protein sequences. The results indicated that VAOTV-1 exhibits low amino acid sequence identity (19%-46%) to all currently recognized viruses (**Figure 2b**). Representative sequences of members of the families *Orthototiviridae* and *Pseudototiviridae*, including well-characterized protozoan-associated viruses such as *Trichomonasvirus* and *Leishmaniavirus* that have been reported from human-derived environments (Goodman et al., 2011), were included in the phylogenetic analysis. Multiple sequence alignments were performed using the MUSCLE algorithm implemented in MEGA-12. The best-fit amino acid substitution model for the RdRp protein was identified as Q.pfam+I+G4. A phylogenetic tree was inferred using the maximum-likelihood method with 1000 bootstrap replicates. Notably, RdRp-based phylogenetic analysis revealed that VAOTV-1 clustered most closely with a rodent-associated totivirus, forming a well-supported clade with bootstrap values exceeding 85% (**Figure 3**). RdRp-based phylogenetic reconstruction resolved members of *Orthototiviridae* and *Pseudototiviridae* into two distinct and strongly supported clades (**Figure 3**). This separation was further corroborated by differences in the consensus sequences of the conserved RdRp motifs (A, B, and C), which exhibited family-specific signature patterns (**Figure 2c**). Sequence logo analysis revealed that although the core catalytic residues of motif C, including the invariant GDD triad, were conserved across all genera, the flanking amino acid residues differed markedly between *Orthototiviridae* and *Pseudototiviridae*. Similar family-level distinctions were observed in motifs A and B. In motif A, members of *Orthototiviridae* typically exhibited a consensus pattern resembling xxxDxxxFDxxxx, whereas *Pseudototiviridae* genera more frequently displayed a xxxDxxDFNSxxx-like signature, reflecting differences in the arrangement of conserved residues surrounding the catalytic aspartate. In motif B, *Orthototiviridae* were characterized by a pattern approximating GxxSGxxxTxxxxxxxN, while *Pseudototiviridae* more commonly showed a TxxSGHRxTTxxxxxLN-like configuration.

To determine whether VAOTV-1 was present at the individual level, nested PCR screening was performed on all 12 original vaginal swab samples. Among these specimens, only one sample tested positive for VAOTV-1 (**Supplementary Figure 1**). The positive sample was obtained from a 16-year-old female patient who was diagnosed with vulvovaginal candidiasis at her initial clinical visit.

### Structural and electrostatic analysis of the VAOTV-1 capsid protein

A structural model of the VAOTV-1 capsid protein was generated using AlphaFold3 (Abramson et al., 2024) (https://alphafoldserver.com/) and visualized with UCSF ChimeraX version 1.9 (Meng et al., 2023). The predicted structure can be broadly divided into two major domains based on secondary structural organization and spatial clustering. In total, the model comprises 15 α-helices (α1–α15) and 5 β-strands (β1–β5). Electrostatic surface analysis of the predicted capsid protein revealed a heterogeneous distribution of charged regions across the surface. Although localized negatively charged patches were observed, the model did not exhibit strong electrostatic polarization at the monomer level. Nevertheless, the presence of discrete acidic regions may be compatible with genome-capsid interactions following higher-order capsid assembly (**Supplementary Figure 2**).

### Detection of VAOTV-1 in public metagenomic datasets

To assess the presence of VAOTV-1 in publicly available vaginal metagenomic datasets, a total of 90 sequencing libraries were analyzed, including 47 paired-end libraries and 43 single-end libraries. Quality-filtered sequencing reads were mapped to the VAOTV-1 reference genome using Bowtie2 in end-to-end mode with the “very-sensitive” preset. Paired-end and single-end libraries were processed according to their respective sequencing layouts. The resulting alignments were converted to sorted BAM files using SAMtools (Danecek et al., 2021). For each library, the total number of sequencing reads and the number of reads mapped to the VAOTV-1 genome were calculated. Only alignments with a mapping quality score ≥30 were retained as high-confidence matches. Genome coverage breadth was defined as the proportion of genomic positions covered by at least one read, and mean sequencing depth was calculated across the entire reference genome. A library was considered positive for VAOTV-1 only when at least three high-confidence reads (mapping quality ≥30) were detected, with genome coverage breadth ≥1% and a mean sequencing depth ≥0.1×. Under these predefined criteria, no evidence of VAOTV-1 was identified in any of the analyzed libraries.

## Discussion

In recent years, the discovery of novel viruses has increased substantially, largely driven by the application of metagenomic sequencing approaches (Mostafa et al., 2020). These methods have enabled the identification of viral sequences in a variety of human and animal tissues, including the intestine, respiratory tract, skin, and blood (Aggarwala et al., 2017; Hannigan et al., 2017). As a result, genomic investigations have uncovered numerous previously uncharacterized viruses that were not detectable using conventional techniques. Although some of these newly identified viruses have been associated with disease development, many appear to cause no obvious harm to their hosts. Clarifying the biological significance of these viruses and their interactions with human or animal hosts is therefore important for determining their potential implications for disease prevention and management.

Toti-like viruses have been increasingly identified across a wide range of hosts, including arthropods such as mosquitoes (Zhai et al., 2010; Isawa et al., 2011) and fruit flies (Wu et al., 2010), as well as bats (Yang et al., 2012) and several fish species (Lovoll et al., 2010). These findings indicate that toti-like viruses may be broadly distributed across diverse ecological niches and host taxa (Leal et al., 2023). In human-derived samples, several totivirus-related viruses have previously been reported, most of which are known to infect protozoan parasites such as *Leishmania*, *Giardia*, and *Trichomonas*. The detection of these viruses is generally associated with the presence of their protozoan hosts and is not considered to reflect direct infection of human cells.

In this study, we identified a novel totivirus-related virus, designated VAOTV-1, from vaginal swab samples collected from patients with vulvovaginal candidiasis. Phylogenetic analysis based on RdRp sequences demonstrated that VAOTV-1 is highly divergent from previously characterized protozoan-associated totiviruses and occupies a distinct lineage. Consistent with its phylogenetic position, VAOTV-1 exhibited low amino acid sequence identity to currently recognized members of the family. Based on genome organization, conserved RdRp motif architecture, sequence identity comparisons, and maximum-likelihood phylogenetic inference, VAOTV-1 can be placed within the family Orthototiviridae, forming a well-supported and evolutionarily distinct clade. To further support functional annotation of the divergent capsid protein, structural prediction was performed using AlphaFold3. The predicted model revealed a compact, α-helix-rich fold consistent with capsid proteins of dsRNA viruses (Stevens et al., 2021; Maurastoni et al., 2023). Electrostatic surface analysis further demonstrated the presence of discrete acidic regions that may contribute to genome-capsid interactions following capsid assembly. Although structural prediction alone cannot establish definitive function, these features provide additional support for annotating the encoded protein as a capsid shell protein. However, the genome remains near-complete, with the 5’ terminus unresolved, and the biological host has not yet been identified. In accordance with the species demarcation criteria of the International Committee on Taxonomy of Viruses (ICTV), viruses sharing less than 50% amino acid sequence identity in their encoded proteins may be considered to represent distinct species, particularly when detected in different host organisms (Reyes-Proano et al., 2024; Simmonds et al., 2024). Given these considerations, VAOTV-1 is most appropriately regarded as a provisional orthototivirus-like virus pending full genome completion and further biological characterization. Considering its detection context and its phylogenetic distance from known protozoan-associated viruses, VAOTV-1 may represent an incidental component of the vaginal microenvironment, the ecological origin and natural host of which remain to be determined. Importantly, the possibility that VAOTV-1 represents an exogenous contaminant appears unlikely, as sequencing reads mapped evenly across the genome without evidence of localized clustering or abnormal coverage patterns, supporting the authenticity of the assembled viral sequence.

Several methodological considerations should be acknowledged when interpreting these findings. Vaginal swab samples were pooled prior to virome enrichment and sequencing, limiting direct assessment of inter-individual variability at the metagenomic stage. Subsequent nested PCR screening detected VAOTV-1 in only one individual; however, the limited sample size restricts accurate estimation of its prevalence. Furthermore, the absence of a control group without vulvovaginal candidiasis precludes definitive evaluation of any association between VAOTV-1 detection and disease status. Future investigations incorporating individual-level sequencing, expanded cohorts, and appropriate comparison groups will be important for clarifying the epidemiological context and potential clinical relevance of this virus.

In summary, this study expands current knowledge of orthototivirus diversity, particularly within human-derived environments. The identification of previously unrecognized eukaryotic viruses in human samples remains relatively uncommon, underscoring the importance of metagenomic approaches for uncovering hidden components of the human virome.

## Data Availability

The datasets presented in this study can be found in online repositories. The names of the repository/repositories and accession number(s) can be found below: https://www.ncbi.nlm.nih.gov/, PRJNA1393300.
