# Supplementary material for: Discovery of a novel orthototivirus-like virus in patients with vulvovaginal candidiasis
Source: Front Cell Infect Microbiol. 2026 Mar 19;16:1779554. doi: 10.3389/fcimb.2026.1779554 (PMC13044131; doi:10.3389/fcimb.2026.1779554)

Supplementary Figure 1. Nested PCR validation of VAOTV-1

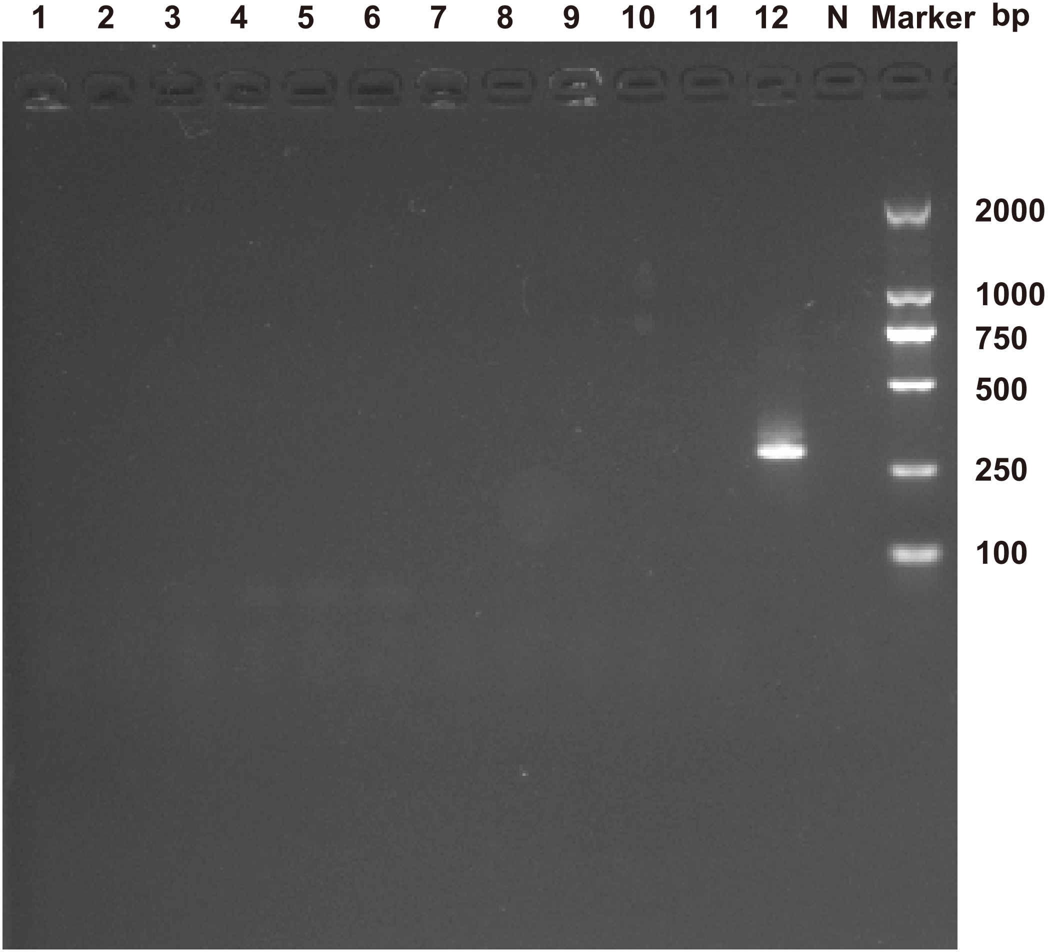

Supplementary Figure 2. AlphaFold3-predicted structural model and electrostatic surface distribution of the VAOTV-1 capsid protein.

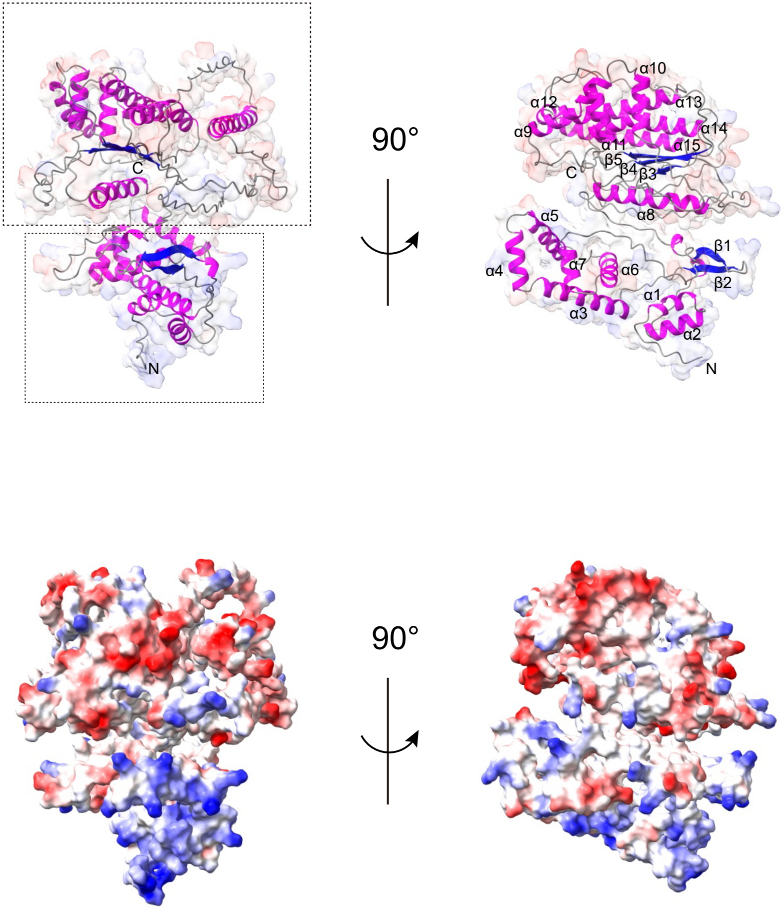

Supplement: Supplementary file 1 [file DataSheet1.pdf]
